# NGR-modified nanovesicles target ALKBH5 to inhibit ovarian cancer growth and metastasis

**DOI:** 10.7150/thno.107766

**Published:** 2025-06-09

**Authors:** Cheng Du, DaLu Wang, Boquan Zhang, Yasong Zhao, Zheng He

**Affiliations:** 1 Department of Obstetrics and Gynecology, Shengjing Hospital of China Medical University, Shenyang 110004, China.; 2 Department of general surgery, Shengjing Hospital of China Medical University, Sanhao Street, Heping District, Shenyang 110004, Liaoning, China.; 3 Department of Nursing, Shengjing Hospital of China Medical University, Heping District, Shenyang 110004, Liaoning, People's Republic of China.; 4 Department of Nursing, Shengjing Hospital of China Medical University, Heping District, Shenyang 110004, Liaoning, People's Republic of China.

**Keywords:** NGR-Modified, Biomimetic Nanovesicles, ALKBH5 siRNA, Ovarian Cancer, Immunotherapy Resistance

## Abstract

**Background:** Immunotherapy resistance in ovarian cancer (OC) poses a significant clinical hurdle. This study aims to investigate the potential of NGR-modified biomimetic nanovesicles (NGR-BNVs) for delivering ALKBH5 siRNA to reverse this resistance.

**Methods:** *In vitro* and *in vivo* experiments were conducted to assess the efficiency of NGR-modified nanovesicles in delivering ALKBH5 siRNA. OC cell proliferation was evaluated, and apoptosis induction was measured. A mouse xenograft model was utilized to examine the effects on tumor volume and metastasis. Tumor immune microenvironment (TIME) analysis was performed to determine changes in immune cell proportions and immunomodulatory factors.

**Results:** NGR-modified nanovesicles effectively delivered ALKBH5 siRNA, leading to a significant inhibition of OC cell proliferation and apoptosis induction. Treated groups in the mouse xenograft model exhibited reduced tumor volume and decreased metastatic signals. Analysis of the immune microenvironment revealed an increased proportion of CD8^+^ T cells, reduced Tregs and MDSCs, and notable changes in key immunomodulatory factors.

**Conclusion:** This study highlights the potential of NGR-modified BNVs for overcoming immunotherapy resistance in OC by delivering ALKBH5 siRNA, resulting in modulation of the immune microenvironment and promising therapeutic outcomes.

## Introduction

Ovarian cancer (OC) is one of the most aggressive and deadly malignancies of the female reproductive system, often referred to as the “silent killer” due to its subtle early symptoms, which frequently lead to late-stage diagnosis 1-3. Despite some advancements in traditional treatments such as surgery, chemotherapy, and targeted therapy in recent years, the overall survival rate of OC remains relatively low 4-6. Recurrent OC poses a particularly grave challenge, as most patients experience relapse after initial treatment, and tumors that recur often exhibit resistance to chemotherapy agents 7-9. Immunotherapy, especially the use of immune checkpoint inhibitors, has brought new hope for the treatment of OC 10. However, clinical observations indicate that only a subset of OC patients respond to immunotherapy, with drug resistance severely limiting its clinical efficacy 11. Therefore, exploring new strategies to overcome immunotherapy resistance is currently a major focus and challenge in research 12-14.

The application of nanotechnology in cancer treatment is becoming increasingly widespread, with nanovesicles being an ideal drug delivery vehicle due to their excellent biocompatibility, controllability, and targeting capabilities 15-17. By modifying the surface of nanovesicles with different targeting ligands, precise delivery to specific cells or tissues can be achieved 18, 19. NGR (Asn-Gly-Arg) is a tripeptide sequence that can specifically bind to tumor vascular endothelial cells and has demonstrated excellent targeting ability in various tumor models 20, 21. NGR-modified nanovesicles not only enhance the targeting of drug delivery but also improve drug accumulation and retention at tumor sites by increasing tumor tissue permeability, thereby enhancing therapeutic effects. Additionally, NGR-modified nanovesicles can reduce side effects and improve treatment safety by avoiding nonspecific distribution in normal tissues.

ALKBH5 is an m6A demethylase that regulates gene expression, cell proliferation, and apoptosis by removing m6A modifications from mRNA 22. Recent studies have linked the abnormal expression of ALKBH5 in various tumors to tumorigenesis, progression, and prognosis 23-25. In OC, high ALKBH5 expression is closely associated with the invasiveness and resistance of tumors 26, 27. Specifically, ALKBH5 influences tumor cell proliferation, invasion, and apoptosis by modulating the m6A modification status of key genes involved in tumor growth and metastasis 22. Therefore, inhibiting ALKBH5 expression holds promise for reversing drug resistance in OC and suppressing its growth and metastasis 28, 29. By delivering ALKBH5 siRNA to OC cells, the specific suppression of ALKBH5 expression can exert anti-tumor effects 30. In addition, ALKBH5 regulates the tumor immune microenvironment (TIME) and participates in cancer progression. ALKBH5 is positively correlated with the expression of programmed death ligand 1 and macrophage infiltration, and is associated with immune therapy response. ALKBH5 promotes the secretion of CCL2 and CXCL10, which recruit programmed death ligand 1-positive tumor-associated macrophages, promoting M2 macrophage polarization. The synergy between ALKBH5 and IL-6 secreted by tumor-associated macrophages activates the JAK2/p-STAT3 pathway in cancer cells, promoting the progression of non-small cell lung cancer 31. In head and neck squamous cell carcinoma, ALKBH5 inhibits RIG-1 expression and interferon-α production via the IKKε/TBK1/IRF3 pathway, thereby promoting tumor progression 32. ALKBH5 promotes macrophage fatty acid metabolism and M2 polarization through the upregulation of CPT1A, thereby promoting colorectal cancer development 33.

ITGB1 protein, also known as integrin β1 (ITGB1), is an important cell adhesion molecule that belongs to the integrin family. The ITGB1 protein consists of α and β subunits and participates in the interaction between cells and the extracellular matrix (ECM), thereby regulating cell adhesion, migration, and signal transduction. The ITGB1 gene is located at the 10p11.22 region on the human chromosome and has multiple alternative splice variants. ITGB1 plays an important role in various biological processes, including embryogenesis, hemostasis, tissue repair, and immune response. Additionally, ITGB1 is highly expressed in various cancer tissues, where it promotes cancer metastasis and spread 34-36. RUNX1 negatively regulates the expression of miR-429 by binding to its promoter, targeting ITGB1, and promoting the growth, metastasis, and EMT of oral squamous cell carcinoma 37. Studies have shown that Zinc finger CCHC-type containing 4 (ZCCHC4) upregulates integrin β1 (ITGB1) to promote osteosarcoma progression 38.

The TIME plays a crucial role in the occurrence, development, and treatment of tumors 39-41. Changes in the proportion and function of immune cells such as CD8^+^ T cells, regulatory T cells (Tregs), and myeloid-derived suppressor cells (MDSCs) in the tumor microenvironment directly impact immune evasion and treatment response in tumors 42-44. CD8^+^ T cells are the main effector cells against tumors and enhancing their quantity and function contributes to improving the efficiency of immune clearance in tumors 45, 46. Conversely, the increase of immune inhibitory cells like Tregs and MDSCs suppresses anti-tumor immune responses, promoting tumor growth and metastasis 47, 42, 48. By modulating key immune factors in the TIME, such as IL-6, TNF-α, and IFN-γ, significant enhancement of anti-tumor immune responses and improved treatment effects can be achieved 49, 50. Therefore, investigating how to regulate the TIME to enhance anti-tumor immune responses is a crucial direction in current cancer treatment research 51-53.

This study aims to reverse immunotherapy resistance in OC by delivering ALKBH5 siRNA using NGR-modified biomimetic nanovesicles (BNVs) and to explore its regulatory mechanism on ITGB1 m6A modification and its impact on the TIME. Specifically, the study first prepared and characterized NGR-modified BNVs, verifying their efficiency and safety in delivering ALKBH5 siRNA. Through *in vitro* and *in vivo* experiments, the study evaluated their effects on OC cell proliferation and apoptosis, as well as their inhibitory effects on tumor growth and metastasis. Simultaneously, the study delved into their regulatory effects on the TIME, including their impact on the proportions of CD8^+^ T cells, Tregs, and MDSCs and the modulation of immune factor expression levels such as IL-6, TNF-α, and IFN-γ. This research not only provides new insights and methods to overcome immunotherapy resistance in OC but also offers a theoretical basis and practical foundation for clinical applications, holding significant scientific and clinical implications. Through this innovative treatment strategy, it is expected to provide more effective therapeutic options for OC patients, thereby enhancing their survival rates and quality of life.

## Materials and Methods

### Part 1: Preparation and Characterization of NGR-Modified BNVs

#### Preparation of Nanovesicles Using Ultrasonication and Extrusion Techniques

Lecithin (Sigma-Aldrich, USA) and cholesterol (Sigma-Aldrich, USA) were dissolved in chloroform at a 1:1 molar ratio and supplemented with 0.1% (v/v) trifluoroacetic acid as a delipidating agent. The solution was subjected to rotary evaporation at 40 °C using a Buchi R-300 rotary evaporator (Switzerland) to form a uniform lipid film. The lipid film was dried in a vacuum desiccator for 2 h to ensure complete solvent removal. Subsequently, the film was dissolved in preheated PBS buffer at 60 °C and sonicated for 20 min in an ultrasonic cleaner (Branson Ultrasonics, USA) to generate primary nanovesicles. To achieve homogenous nanovesicles, the primary nanovesicles were extruded 21 times through a 100 nm polycarbonate membrane (Whatman Nuclepore, USA) using an extrusion device (Avanti Polar Lipids, USA) to ensure uniform particle size distribution.

#### Co-loading of ALKBH5 siRNA

During the preparation of nanovesicles, ALKBH5 siRNA (Thermo Fisher Scientific, USA) was co-treated with a mixture of phospholipids and cholesterol to encapsulate it within the nanovesicles. The nanovesicles loaded with siRNA were separated using the ethanol precipitation method. The specific procedure is as follows: the nanovesicle solution was mixed with ethanol at a ratio of 1:4 by volume, left undisturbed for 15 min, centrifuged at 10,000 g for 10 min, the supernatant was removed, and the precipitate was collected. The precipitate was dissolved in PBS buffer, and the drug loading of siRNA in the nanovesicles was quantitatively detected using fluorescence quantitative Polymerase Chain Reaction (PCR) (Applied Biosystems QuantStudio 5, USA) to assess the encapsulation efficiency and quantify the amount of siRNA. Encapsulated siRNA in the nanovesicles remained trapped in the gel pores, while free siRNA formed bands in the gel during electrophoresis, resulting in the separation of free siRNA from the nanovesicles. The encapsulation efficiency was calculated as (Ctotal - Cfree) / Ctotal × 100%.

#### Chemical Crosslinking Modification of NGR Peptide

In this study, the NGR peptide (obtained from Peptide 2.0, USA) was chemically crosslinked to the surface of nanocapsules through an amine reaction. The specific procedure involved incubating NGR peptide (1 mg/mL) with EDC (1-ethyl-3-(3-dimethylaminopropyl) carbodiimide) and NHS (N-hydroxysuccinimide) (obtained from Thermo Fisher Scientific, USA) in a molar ratio of 1:2:2 in PBS buffer (pH 7.4) at room temperature for 30 min. Subsequently, the reaction solution was added to the nanocapsule suspension and allowed to react for 2 h at room temperature. The modification efficiency of NGR peptide was evaluated using UV-visible spectrophotometry (Thermo Fisher Scientific Nanodrop 2000, USA), and the binding of NGR peptide was confirmed by high-performance liquid chromatography (HPLC, Agilent Technologies 1260 Infinity II, USA).

#### Characterization of Nanovesicles

Nanovesicle size and distribution were measured using the Zetasizer Nano ZS instrument (Malvern Panalytical, UK). Prior to each measurement, the nanovesicle samples were diluted to 1 mg/mL in deionized water and incubated at 37 °C for 30 min to mimic physiological conditions. The measured data included average size, polydispersity index (PDI), and zeta potential to evaluate the stability of nanovesicles under physiological conditions. The morphology of nanovesicles was observed using transmission electron microscopy (TEM) (JEOL JEM-2100, Japan). For sample preparation, the nanovesicle solution was drop-cast onto a copper grid, air-dried, and then negatively stained with 1% phosphotungstic acid (PTA). Subsequently, the samples were examined under TEM to confirm their morphology and structure.

Furthermore, the nanovesicle samples underwent 1% agarose gel electrophoresis, and the encapsulation of siRNA was detected using a UV lamp (UVP UV Transilluminator, USA). Fluorescence quantitative PCR (Applied Biosystems QuantStudio 5, USA) was employed to quantitatively measure the drug loading of siRNA in nanovesicles. The specific procedure involved diluting the nanovesicle solution to an appropriate concentration, taking a certain volume of the sample for PCR amplification, and recording and analyzing the fluorescence signal intensity to determine the amount of siRNA loaded.

To facilitate subsequent observation and analysis, both NGR-BNVs and BNVs were fluorescently labeled using the lipophilic dye DiO (Fubio, Cat. No. 22046), which integrates into the lipid bilayer of the vesicles. To verify the successful conjugation of the NGR peptide to the surface of BNVs and to calculate the conjugation efficiency, high-performance liquid chromatography (HPLC) was used to detect the amount of free NGR peptide. After the modification reaction, the product was processed using a 10 kDa ultrafiltration centrifuge tube (Millipore), and the filtrate was collected for HPLC analysis. The detection wavelength was set at 214 nm. The retention time of the NGR peptide was approximately 13.2 minutes. The peak area of the NGR peptide was compared with a standard curve to determine the residual concentration of free NGR after the reaction. The conjugation efficiency was calculated using the following formula:



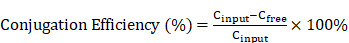



### Part 2: *In Vitro* Cellular Experiments

#### OC Cell Culture and Group Interventions

The OC cell lines SKOV3 and OVCAR-3 (obtained from ATCC, USA) were cultured in RPMI-1640 medium (Gibco, USA) containing 10% fetal bovine serum (Gibco, USA) at 37 °C in a 5% CO₂ humidified incubator. The culture medium was regularly replaced to maintain cells in the logarithmic growth phase.

The prepared nanovesicles were divided into six groups for cell treatment: control group, BNVs group, NGR-modified nanovesicle group, ALKBH5 siRNA nanovesicle group, and NGR-modified ALKBH5 siRNA nanovesicle group, NGR-modified ALKBH5 siRNA nanovesicle group, and NGR-modified ALKBH5 siRNA nanovesicle + ITGB1 overexpression group. Subsequent experimental analyses were conducted after treating each cell group.

#### Cell Proliferation and Apoptosis Detection

Cell proliferation was assessed using the CCK-8 assay kit (Dojindo, Japan). Cells from each group were seeded in a 96-well plate, and after the addition of CCK-8 reagent, absorbance at 450 nm was measured. Three replicates were performed for each group, with the experiment repeated three times.

Furthermore, apoptosis detection was conducted using the Annexin V-FITC/PI apoptosis detection kit (BD Biosciences, USA). Cells were digested with trypsin, centrifuged, resuspended in Annexin V binding buffer, stained with Annexin V-FITC and PI, and analyzed for apoptotic cell proportion using flow cytometry. The experiment was repeated three times.

#### Cell Immunotherapy Resistance Testing

OC cells were co-cultured with T cells at different ratios (e.g., 1:1, 2:1) for 48 h. The apoptosis and proliferation of co-cultured cells were analyzed using CCK-8 and flow cytometry. The supernatant from co-culture was collected, and the secretion levels of IFN-γ and TNF-α were detected using Western Blot. The experiment was repeated three times.

#### Analysis of Molecular Biology Techniques

For gene expression analysis using RT-qPCR, total RNA was extracted from cells and reverse transcribed into cDNA using the RT-qPCR kit (Takara, Japan). The expression changes of ITGB1 and ALKBH5 genes were then detected using the SYBR Green qPCR kit on a real-time quantitative PCR instrument. The experiment was repeated three times.

To detect protein expression via Western Blot, total cellular proteins were extracted, separated by SDS-PAGE electrophoresis, transferred to membranes, and probed with specific antibodies (Cell Signaling Technology, USA). Protein expression levels were visualized using ECL detection, and the experiment was repeated three times.

Immunofluorescence staining involved fixing treated cells on glass slides and performing immunofluorescence staining with specific antibodies against ALKBH5 and ITGB1 (Cell Signaling Technology, USA). The cellular localization and interaction of the proteins were observed using laser confocal microscopy, with the experiment repeated three times.

### Part 3: *In vivo* Animal Experiments

#### Ethical Statement

The animal experiments have been approved by the Institutional Animal Ethics Committee. The breeding and handling of all experimental animals strictly adhere to the Regulations on the Management of Laboratory Animals and the Guidelines for the Use of Laboratory Animals, ensuring animal welfare. Throughout the experimental procedures, efforts are made to minimize animal suffering and unnecessary harm, with all operations performed under aseptic conditions. Appropriate anesthetics are used during tumor volume monitoring and bioluminescence imaging, and euthanasia is employed as the humane endpoint for the experiments.

#### *In Vivo* Biocompatibility

Eight-week-old female BALB/c mice (purchased from Beijing Vital River Laboratory Animal Technology Co., Ltd.) were randomly divided into two groups, with 6 mice in each group. The experimental group was intravenously injected with NGR-modified ALKBH5 siRNA nanovesicles (NGR-ALKBH5-siRNA-BNVs) at a dose of 10 mg/kg, while the control group was injected with an equal volume of saline. Mouse body weight was monitored every two days for 14 days. After the experiment, blood was collected from the orbital vein for routine blood tests and biochemical indicators. The routine blood analysis was performed using an automatic blood cell counter (Mindray BC-5000, China), including leukocyte, erythrocyte, and platelet counts. Biochemical indicators aspartate transaminase, alanine transaminase, blood urea nitrogen, and creatinine (ALT, AST, BUN, Cr) were measured using an automatic biochemical analyzer (Hitachi 7180, Japan). The major organs (heart, liver, spleen, lungs, kidneys) were fixed in 4% paraformaldehyde, embedded in paraffin, sectioned (5 µm), stained with HE, and observed under a microscope (Nikon Eclipse E100, Japan) for histopathological changes 54.

#### Establishment of Subcutaneous Xenograft Mouse Model

Six to eight-week-old NOD/SCID immunodeficient mice were obtained from Beijing Vital River Laboratory Animal Technology Co., Ltd. This strain was chosen for its highly immunodeficient state, which is ideal for human tumor cell transplantation, as previous studies have confirmed the high tumor engraftment efficiency of NOD/SCID mice in cancer research. The mice were housed in aseptic conditions with *ad libitum* access to food and water, following a 12-hour light/dark cycle. Log-phase SKOV3 OC cells (ATCC, USA) were suspended in pH 7.4 PBS and adjusted to a concentration of 2×10^6^ cells/100 μL. Subcutaneous injections of 100 μL of cell suspension were administered on the right dorsal side of each mouse using a sterile syringe. Once the tumor volume reached approximately 100 mm³, the mice were randomly divided into 6 groups of 10 each group. Subsequently, each group was injected with 5×10⁶ cells/100 μL of human T cells (isolated from peripheral blood donated by healthy volunteers).

The interventions for each group were as follows: (1) Control group (no treatment), NGR-BNVs group: Mice were intravenously injected with NGR-BNVs (0.5 mg/kg) twice a week for 4 weeks. (2) BNVs group: Mice were intravenously injected with BNVs (0.5 mg/kg) twice a week for 4 weeks. (3) NGR-BNVs group: Mice were intravenously injected with NGR-BNVs (0.5 mg/kg) twice a week for 4 weeks. (4) ALKBH5-siRNA-BNVs group: Mice were intravenously injected with ALKBH5-siRNA-BNVs (0.5 mg/kg) twice a week for 4 weeks. (5) NGR-ALKBH5-siRNA-BNVs group: Mice were intravenously injected with NGR-ALKBH5-siRNA-BNVs (0.5 mg/kg) twice a week for 4 weeks. (6) NGR-ALKBH5-siRNA-BNVs + ITGB1 group: Mice were intravenously injected with NGR-modified biomimetic nanovesicles loaded with ALKBH5 siRNA (0.5 mg/kg) twice a week for 4 weeks, in combination with intravenous injection of the ITGB1 overexpression vector (Vigene Biosciences, China) (1×10⁸ TU), twice a week for 4 weeks. This dosage was based on previous dose-effect experiments, which showed that this concentration effectively promoted tumor suppression with no significant toxic side effects.

#### Nanovesicle Tracking in Mice

Fluorescently or radioactively labeled NGR-BNVs and BNVs were intravenously injected into tumor-bearing mice at a dose of 0.5 mg/kg. Following injection, imaging and analysis were conducted at time points of 1, 2, 4, 8, 12 and 24 hours using an *in vivo* imaging system. The distribution of the nanovesicles within the tumor and other organs was observed by assessing fluorescence or radioactive signals, and the accumulation levels in tumor tissues were evaluated.

#### Detection of Tumor Growth and Metastasis

Tumor dimensions were measured every three days using a caliper to determine Length and width, and the volume was calculated using the formula: Volume = Length × Width²/2. Additionally, tumor growth and metastasis within the mice were monitored once a week in an anesthetized state following injection of D-luciferin potassium salt (GoldBio, USA) using the IVIS Spectrum imaging system (PerkinElmer, USA).

#### Analysis of TIME

Initially, tumor tissues were collected and enzymatically digested to prepare single-cell suspensions. Cell immunostaining was performed using a panel of antibodies, specifically including CD45 (clone: HI30), CD3 (clone: HIT3a), CD4 (clone: RPA-T4), CD8 (clone: RPA-T8), FoxP3 (clone: 259D/C7), and Gr-1 (clone: RB6-8C5), all antibodies were provided by BD Biosciences, USA. Cell analysis was conducted using the BD LSR Fortessa X-20 flow cytometer, and data acquisition and analysis were performed using BD FacSuite software. Total RNA from tumor tissues was extracted using Trizol reagent (ThermoFisher Scientific, USA) and reverse transcribed into cDNA using the Reverse Transcriptase Kit (ThermoFisher Scientific, USA). Real-time quantitative PCR analysis was carried out using SYBR Green PCR Master Mix (ThermoFisher Scientific, USA) to detect the mRNA expression levels of immune regulatory factors such as IL-6, TNF-α, and IFN-γ, with all primers supplied by Sangon Biotech (China). Furthermore, total proteins from the tumor tissues were extracted using RIPA lysis buffer (Beyotime, China), separated by SDS-PAGE electrophoresis, transferred onto membranes, and subjected to Western Blot analysis using specific antibodies (such as IL-6, TNF-α, IFN-γ, Abcam, USA), followed by visualization using ECL reagent (ThermoFisher Scientific, USA).

### Statistical Analysis

All data in this study were statistically analyzed using GraphPad Prism (GraphPad Software, USA) or SPSS (IBM, USA) software. Tumor volume data was compared among different time points and groups using repeated measures analysis of variance (ANOVA); the proportions of immune cells between groups and the levels of gene and protein expression were analyzed through one-way ANOVA and post hoc analysis was conducted using Tukey's multiple comparison test. For non-normally distributed data, the Mann-Whitney U test was employed. Experimental data are presented as mean ± standard deviation (Mean ± SD), with statistical significance set at *p* < 0.05. These statistical methods were essential to ensure the accuracy of data analysis and the reliability of the results.

## Results

### Precise Drug Delivery Achieved using NGR-Modified BNVs

**Figure 1:**
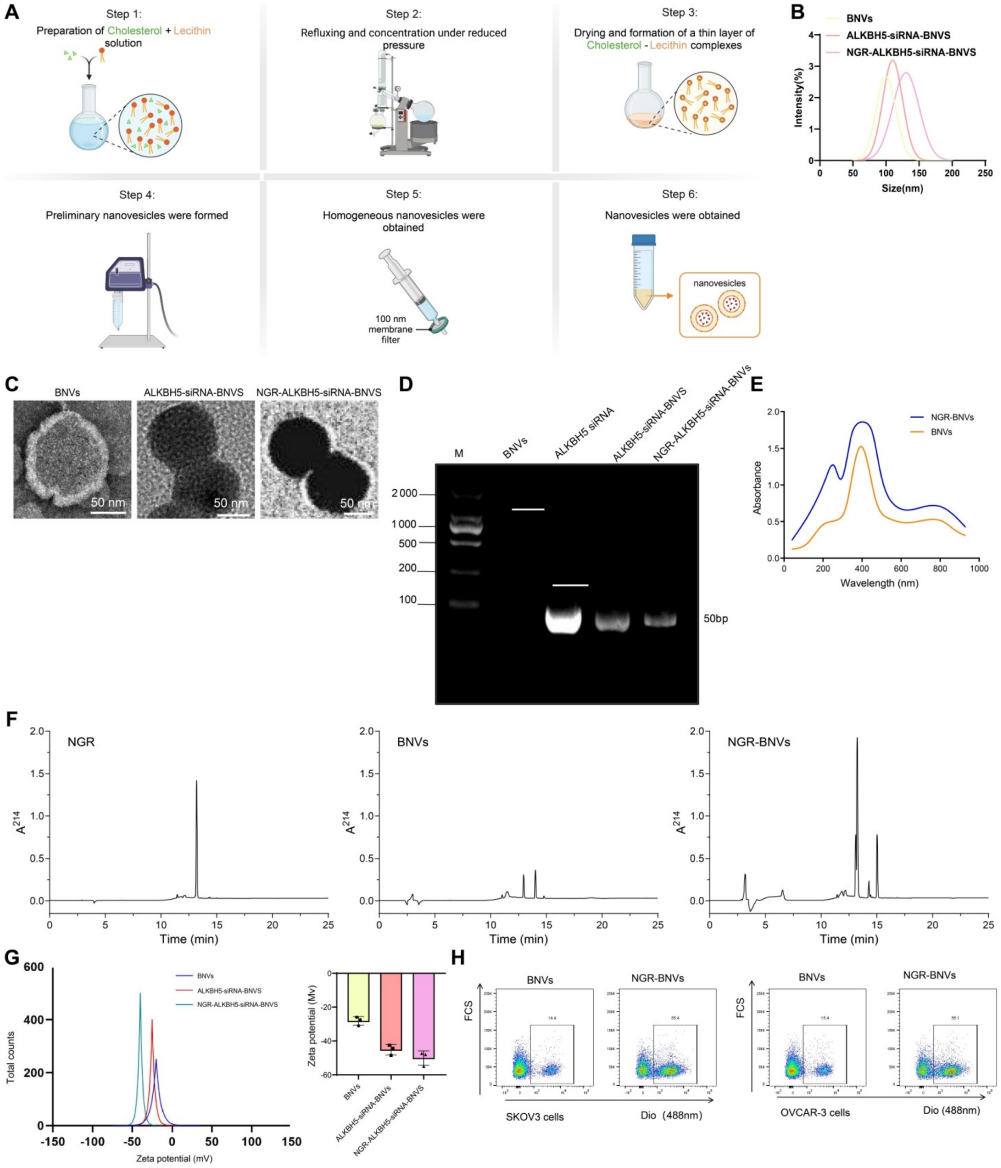
** Preparation and Characterization of NGR-Modified BNVs.** Note: (A) Schematic diagram of the preparation process of NGR-ALKBH5-siRNA-BNVs (BNVs: biomimetic nanovesicles; ALKBH5-siRNA-BNVs: biomimetic nanovesicles encapsulating ALKBH5 siRNA; NGR-ALKBH5-siRNA-BNVs: NGR-modified biomimetic nanovesicles encapsulating ALKBH5 siRNA); (B) Particle size distribution of BNVs, ALKBH5-siRNA-BNVs, and NGR-ALKBH5-siRNA-BNVs; (C) Transmission electron microscope (TEM) images of BNVs, ALKBH5-siRNA-BNVs, and NGR-ALKBH5-siRNA-BNVs (scale bar: 50 μm); (D) Fluorescence qPCR results of ALKBH5@BNVs; (E) UV-visible spectrophotometry analysis of the absorbance values of NGR peptide and NGR-BNVs (NGR peptide exhibits an absorption peak at a specific wavelength, which, combined with a standard curve, enables calculation of peptide concentration and modification efficiency); (F) HPLC chromatograms showing characteristic peaks of NGR peptide, BNVs, and NGR-BNVs; (G) Zeta potential analysis of BNVs, ALKBH5-siRNA-BNVs, and NGR-ALKBH5-siRNA-BNVs; (H) Flow cytometry analysis of drug accumulation and targeting capability of BNVs and NGR-BNVs in SKOV3 and OVCAR-3 cells. All experiments were repeated three times.

Initially, we successfully prepared BNVs through sonication and extrusion methods (Figure 1A). Dynamic light scattering (DLS) results demonstrated that the average size of the prepared BNVs was 120 ± 10 nm, with a PDI below 0.2, indicating good uniformity and stability of the nanovesicles (Figure 1B). TEM images revealed a well-rounded and smooth morphology, indicating an intact and uniform structure of the prepared nanovesicles (Figure 1C).

ALKBH5 is an RNA demethylase enzyme that, upon inhibition, reduces the m6A modification levels in tumor cells, thereby affecting ITGB1 expression and inhibiting tumor cell proliferation and metastasis. To effectively silence ALKBH5 gene expression, reverse tumor cell resistance, and enhance immunotherapy efficacy, we encapsulated ALKBH5 siRNA within the nanovesicles to obtain ALKBH5-siRNA-BNVs. Quantification of siRNA loading in the vesicles using fluorescent quantitative PCR showed that each milligram of nanovesicles carried 0.5 micrograms of siRNA, with an encapsulation efficiency of over 85%, confirming effective siRNA encapsulation within the nanovesicles (Figure 1D). Consistent siRNA loading and encapsulation efficiency were observed across multiple repeat experiments, indicating the reproducibility and stability of the preparation method.

NGR peptide, a tumor vasculature-targeting peptide, specifically binds to aminopeptidase N (CD13) on the surface of tumor neovascular endothelial cells, enabling targeted delivery. To enhance the tumor-targeting capability of the nanovesicles, we successfully modified the NGR peptide onto the surface of BNVs using EDC/NHS crosslinking, resulting in NGR-ALKBH5-siRNA-BNVs. UV-visible spectrophotometry revealed a modification efficiency of 75% for the NGR peptide, demonstrating a high modification efficiency (Figure 1E). UV-Vis spectrophotometry was performed in the wavelength range of 200-800 nm, and the spectra of the NGR peptide blank group and NGR-ALKBH5-siRNA-BNVs experimental group were recorded. A new peak should appear at the characteristic wavelength of the marker (260 nm) in the NGR-ALKBH5-siRNA-BNVs experimental group, with the absorbance at the corresponding wavelength significantly higher than that of the blank group (Figure 1E), confirming that the NGR peptide successfully bound to the surface of the nanovesicles. Meanwhile, HPLC analysis further confirmed the binding of the NGR peptide, showing distinct characteristic spectral peaks for NGR peptide, BNVs, and NGR-BNVs, demonstrating that not only did the NGR peptide successfully bind to the surface of the nanovesicles, but the modification process was also stable and controllable (Figure 1F).

Subsequently, the average size of NGR-modified nanovesicles was measured using Zetasizer Nano ZS, indicating an average size of 130 ± 12 nm with a PDI of 0.18, showcasing good uniformity and stability (Figure 1B). Zeta potential analysis revealed a surface potential of -25 mV for the NGR-modified nanovesicles, indicating good dispersion and stability in solution (Figure 1G). Additionally, TEM images showed that the NGR-modified nanovesicles maintained a well-rounded and smooth surface, further confirming the structural integrity and uniformity of the nanovesicles (Figure 1C). Gel electrophoresis of NGR-ALKBH5-siRNA-BNVs samples in 1% agarose gel under UV light exhibited well-packed ALKBH5 siRNA within the nanovesicles without any noticeable free siRNA bands, confirming effective siRNA encapsulation within the NGR-modified nanovesicles (Figure 1D). These results were consistent with the fluorescence quantitative PCR results of unmodified nanovesicles, further validating the efficient loading and encapsulation capacity of siRNA within the nanovesicles (Figure 1D). To verify the drug accumulation and targeting, flow cytometry analysis was conducted using BNVs and NGR-BNVs labeled with the green lipophilic dye Dio. The results indicated that NGR-modified nanovesicles significantly increased drug accumulation in target cells, including the ovarian cancer cell lines SKOV3 and OVCAR-3 (Figure 1H).

These experimental findings validate the excellent characteristics of NGR-modified BNVs in terms of size, morphology, surface potential, and siRNA loading, laying a solid foundation for their application in targeted drug delivery for cancer therapy.

### NGR-Modified BNVs Significantly Inhibit OC Cell Proliferation and Induce Apoptosis

**Figure 2:**
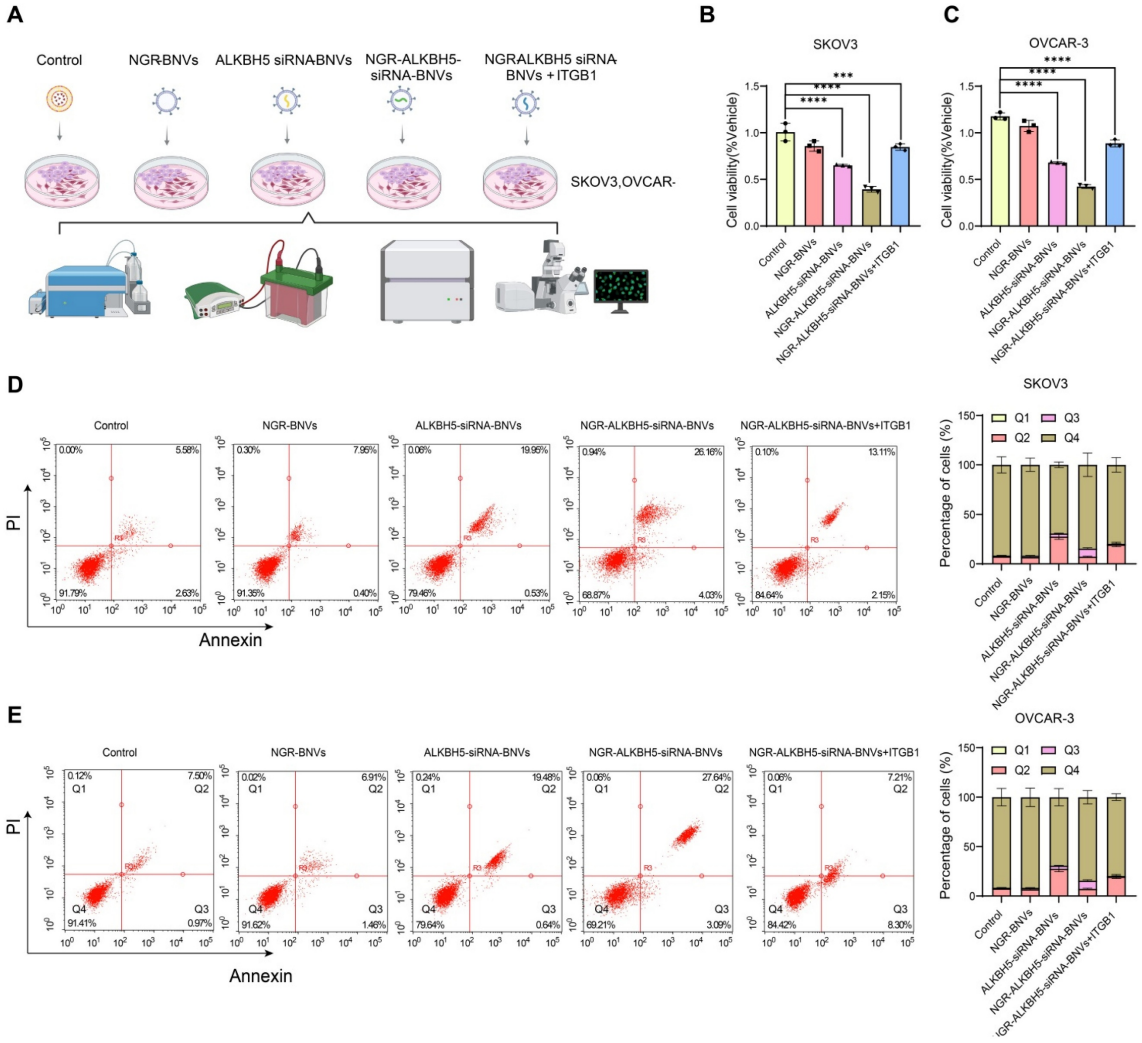
** Impact of NGR-Modified BNVs on OC Cell Proliferation and Apoptosis.** Note: (A) Schematic representation of the process of NGR-modified BNVs treatment of OC cells; (B) CCK-8 assay evaluating the proliferative capacity of SKOV3 cells in different treatment groups. Compared to the control group, both the NGR-modified nanovesicle group and the ALKBH5 siRNA nanovesicle group significantly inhibited cell proliferation, with the NGR-modified ALKBH5 siRNA nanovesicle group showing the most significant inhibitory effect. Rescue experiments assessed the proliferative capacity of SKOV3 cells after treatment with NGR-modified ALKBH5 siRNA nanovesicles. The results indicated that upon the overexpression of ITGB1 plasmids, the cell proliferation capacity partially recovered, suggesting the crucial role of ITGB1 in the modulation of cell proliferation by NGR-modified ALKBH5 siRNA nanovesicles. (C) CCK-8 assay assessing the proliferative capacity of OVCAR-3 cells in different treatment groups. Both the NGR-modified nanovesicle group and the ALKBH5 siRNA nanovesicle group significantly suppressed cell proliferation, with the NGR-modified ALKBH5 siRNA nanovesicle group demonstrating the most pronounced effect. Rescue experiments on OVCAR-3 cells evaluated the proliferative and apoptotic rates after treatment with NGR-modified ALKBH5 siRNA nanovesicles. The addition of ITGB1 overexpressing plasmids led to a partial recovery in cell proliferation capacity, further confirming the critical role of ITGB1. (D) Flow cytometry analysis of apoptosis rates in different treatment groups of SKOV3 cells. The NGR-modified ALKBH5 siRNA nanovesicle group induced the highest apoptosis rate, significantly higher than other treatment groups. Rescue experiments on SKOV3 cells treated with NGR-modified ALKBH5 siRNA nanovesicles showed a significant decrease in apoptosis rate upon the addition of ITGB1 overexpressing plasmids, indicating the important role of ITGB1 in the induction of cell apoptosis by NGR-modified ALKBH5 siRNA nanovesicles. (E) Flow cytometry analysis of apoptosis rates in different treatment groups of OVCAR-3 cells (Q1 represents necrotic cells, Q2 represents late apoptotic cells, Q3 represents early apoptotic cells, and Q4 represents viable cells). Similarly, the NGR-modified ALKBH5 siRNA nanovesicle group induced the highest apoptosis rate, significantly higher than other treatment groups. Rescue experiments on OVCAR-3 cells after treatment with NGR-modified ALKBH5 siRNA nanovesicles demonstrated a significant decrease in apoptosis rate upon the addition of ITGB1 overexpressing plasmids, further validating the critical role of ITGB1. The experiments were repeated three times, and the data in the figures are presented as mean ± SD. ANOVA was used for statistical analysis, with * indicating *p* < 0.05 and *** indicating *p* < 0.001.

Figure 2A depicts the process of NGR-modified BNV treatment on OC cells. The proliferation capacity of OC cell lines SKOV3 and OVCAR-3 was assessed using the CCK-8 assay. The results indicate that, compared to the control group, the NGR-modified nanovesicle group showed no significant difference in cell proliferation. The ALKBH5 siRNA nanovesicle group significantly inhibited cell proliferation. Particularly, the NGR-modified ALKBH5 siRNA nanovesicles exhibited a remarkable decrease in cell viability (Figure 2B-C). Flow cytometry analysis revealed that the NGR-modified ALKBH5 siRNA nanovesicles induced the highest rate of apoptosis, with a significantly greater proportion of apoptotic cells than other treatment groups (Figure 2D-E).

Rescue experiments indicated that upon overexpression of ITGB1 plasmid, the cell proliferation capacity partially recovered, and the apoptosis rate significantly decreased (Figure 2A-B). This suggests that ITGB1 plays a crucial role in the induction of apoptosis by NGR-modified ALKBH5 siRNA nanovesicles. These findings underscore the substantial anti-tumor activity of NGR-modified ALKBH5 siRNA nanovesicles, capable of reducing tumor cell numbers by inhibiting proliferation and inducing apoptosis.

### NGR-Modified ALKBH5 siRNA Nanovesicles Enhance Sensitivity of OC Cells to T Cells

**Figure 3:**
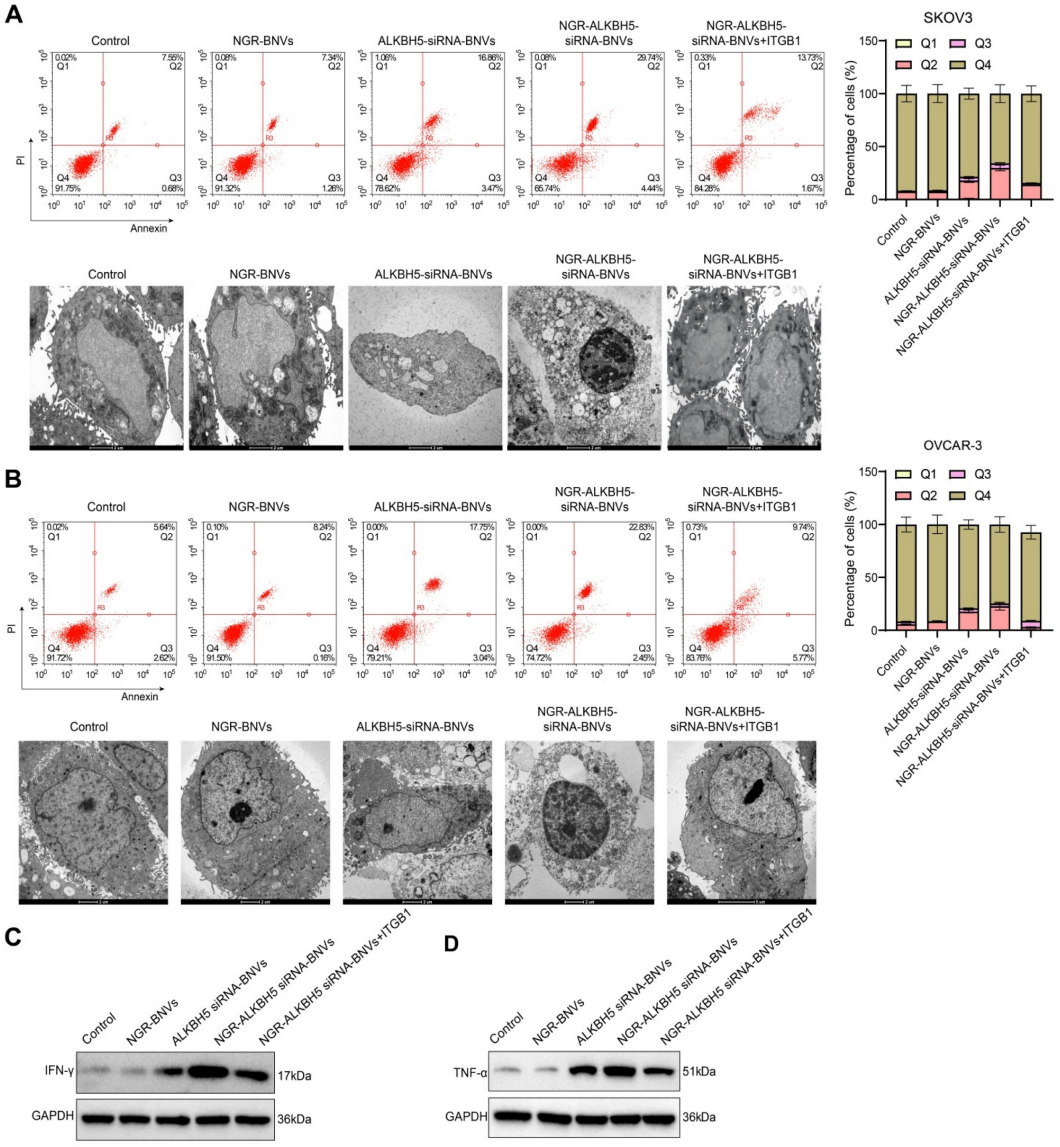
** The Impact of NGR-Modified ALKBH5 siRNA Nanovesicles on Immune Cell Activity Regulating OC Cell Growth.** Note: (A) Differential effects of OC cells in various treatment groups on T cell apoptosis in co-culture experiments. The NGR-modified ALKBH5 siRNA nanovesicle group significantly increased the apoptosis rate of OC cells induced by T cells. The rescue experiment assessed the sensitivity of SKOV3 cells to T cells after treatment with NGR-modified ALKBH5 siRNA nanovesicles. Partial restoration of OC cell sensitivity to T cells was observed upon transfection with PD-L1 overexpressing plasmid, indicating the crucial role of ITGB1 in immune evasion. Electron microscopy images showed apoptotic cellular morphology: NGR-modified ALKBH5 siRNA nanovesicles notably intensified the apoptotic morphology of OC cells induced by T cells, including cell shrinkage, chromatin condensation, and formation of apoptotic bodies. (B) The effects of different treatment groups of OC cells on T cell proliferation inhibition in co-culture experiments. The NGR-modified ALKBH5 siRNA nanovesicle group significantly inhibited the proliferation of OC cells. The rescue experiment evaluated the sensitivity of OVCAR-3 cells to T cells after treatment with NGR-modified ALKBH5 siRNA nanovesicles. Partial restoration of OC cell sensitivity to T cells was observed upon transfection with ITGB1 overexpressing plasmid, further confirming the critical role of ITGB1. Electron microscopy of apoptotic morphology: The NGR-modified ALKBH5 siRNA nanovesicle group significantly exacerbated the T cell-induced apoptotic morphology of OC cells, including cell shrinkage, chromatin condensation, and apoptotic body formation. (C) Western blot analysis of IFN-γ secretion levels in the supernatant of co-culture in different treatment groups. The NGR-modified ALKBH5 siRNA nanovesicle group significantly increased the secretion of IFN-γ. (D) Western blot analysis of TNF-α secretion levels in the supernatant of co-culture in different treatment groups. The NGR-modified ALKBH5 siRNA nanovesicle group significantly increased the secretion of TNF-α (Q1 represents necrotic cells, Q2 represents late apoptotic cells, Q3 represents early apoptotic cells, Q4 represents viable cells). All experiments were repeated three times, and the data in the figures are presented as mean ± SD. ANOVA was used for statistical analysis, with * indicating *p* < 0.05 and *** indicating *p* < 0.001.

Cell coculture experiments were conducted to assess the changes in the sensitivity of OC cells to T cells after treatment with nanovesicles. The results showed that the NGR-modified ALKBH5 siRNA nanovesicle group significantly increased OC cell sensitivity to T cells. Compared to the NGR-ALKBH5 siRNA-BNVs group, the NGR-ALKBH5 siRNA-BNVs + ITGB1 group showed a marked reduction in OC cell sensitivity to T cells (Figure 3A-B). Western blotting results showed that, compared to the control group, the NGR-modified ALKBH5 siRNA nanovesicle group significantly increased the secretion levels of IFN-γ and TNF-α in the co-culture supernatant, indicating that this treatment effectively activated the anti-tumor activity of immune cells. In contrast, the secretion levels of IFN-γ and TNF-α were significantly reduced in the NGR-ALKBH5 siRNA-BNVs and NGR-ALKBH5 siRNA-BNVs + ITGB1 groups (Figure 3C-D).

These findings indicate that NGR-modified ALKBH5 siRNA nanovesicles not only directly inhibit tumor cell proliferation but also suppress tumor cell growth further by augmenting immune cell activity.

### NGR-Modified ALKBH5 siRNA Nanovesicles Regulate the Malignant Biological Behavior of OC Cells by Modulating ITGB1 and ALKBH5 Gene and Protein Expression

**Figure 4:**
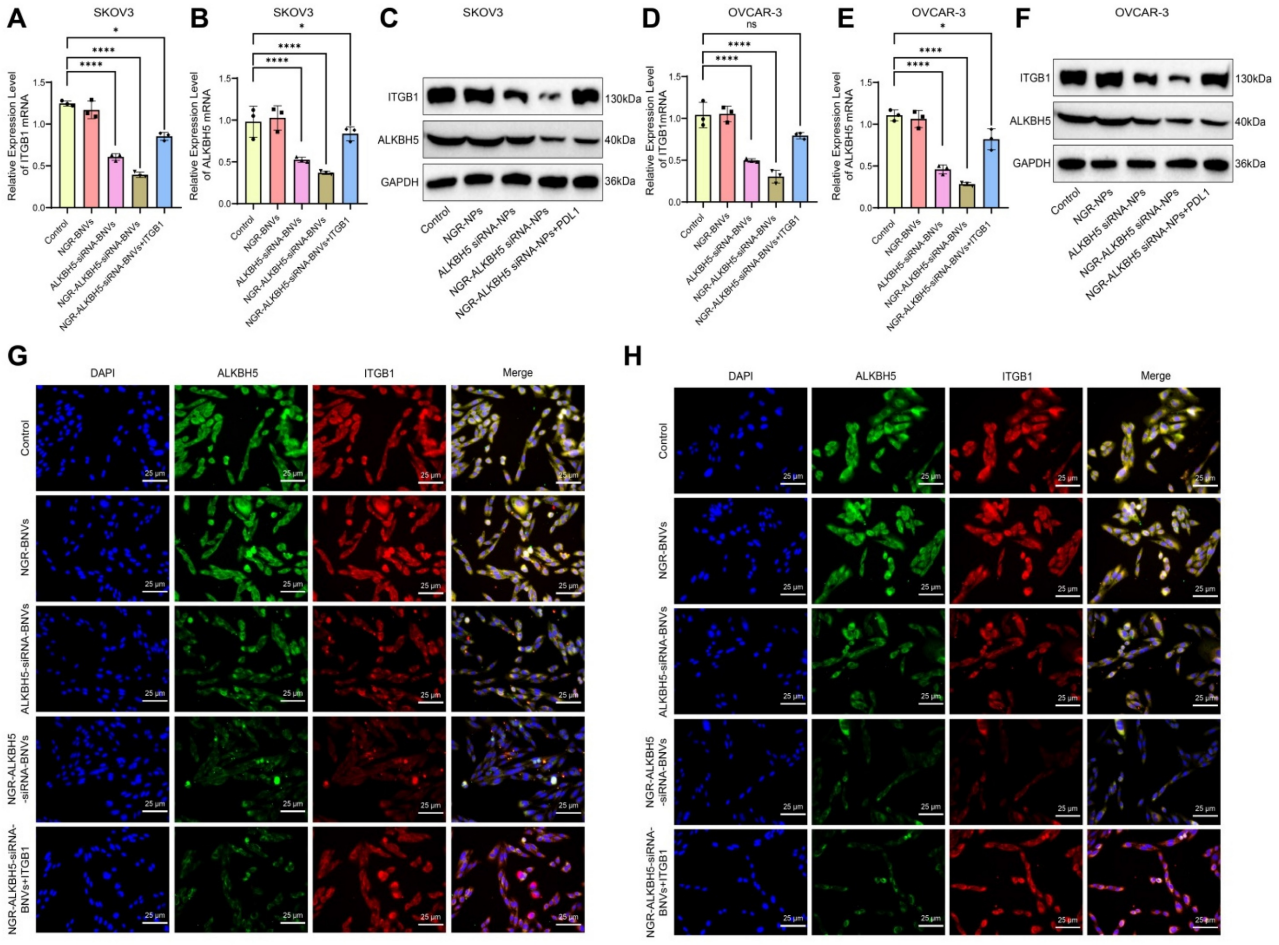
** Regulation of ITGB1 and ALKBH5 Gene and Protein Expression by NGR-Modified ALKBH5 siRNA Nanovesicles.** Note: (A) RT-qPCR analysis of ITGB1 gene expression in SKOV3 cells; (B) RT-qPCR analysis of ALKBH5 expression in SKOV3 cells; (C) Western blot analysis of ITGB1 and ALKBH5 protein expression in SKOV3 cells; (D) RT-qPCR analysis of ITGB1 gene expression in OVCAR-3 cells; (E) RT-qPCR analysis of ALKBH5 gene expression in OVCAR-3 cells; (F) Western Blot analysis of ITGB1 and ALKBH5 protein expression in OVCAR-3 cells; (G) Immunofluorescence staining of the co-localization of ALKBH5 and ITGB1 in SKOV3 cells; (H) Immunofluorescence staining in OVCAR-3 cells to assess the co-localization of ALKBH5 and ITGB1 proteins. All experiments were repeated three times, and the data are presented as mean ± SD. Statistical significance was determined by ANOVA, with * indicating *p* < 0.05 and *** indicating *p* < 0.001.

RT-qPCR results showed that, compared to the Control group, the ALKBH5 siRNA-BNVs and NGR-ALKBH5 siRNA-BNVs groups had significantly reduced expression of ITGB1 and ALKBH5 genes. Compared to the NGR-ALKBH5 siRNA-BNVs group, the NGR-ALKBH5 siRNA-BNVs + ITGB1 group showed significantly increased ITGB1 gene expression, while ALKBH5 protein expression did not show significant differences (Figure 4A-B, Figure 4D-E). Western blot analysis further confirmed these results. Compared to the Control group, the ALKBH5 siRNA-BNVs and NGR-ALKBH5 siRNA-BNVs groups showed significantly reduced expression of ITGB1 and ALKBH5 proteins. Compared to the NGR-ALKBH5 siRNA-BNVs group, the NGR-ALKBH5 siRNA-BNVs + ITGB1 group showed significantly increased ITGB1 protein expression, while ALKBH5 protein expression did not show significant differences (Figure 4C, Figure 4F). Immunofluorescence staining results indicated that the intracellular localization of ALKBH5 and ITGB1 underwent significant changes. Compared to the Control group, ITGB1 and ALKBH5 protein expression were significantly reduced in the ALKBH5 siRNA-BNVs and NGR-ALKBH5 siRNA-BNVs groups. Compared to the NGR-ALKBH5 siRNA-BNVs group, ITGB1 protein expression was significantly increased in the NGR-ALKBH5 siRNA-BNVs + ITGB1 group, while ALKBH5 protein expression did not show significant differences (Figure 4G-H).

These results suggest that NGR-modified ALKBH5 siRNA nanovesicles can inhibit the expression of ITGB1.

### NGR-Modified BNVs Efficiently Inhibit Tumor Growth and Metastasis in Mice

The NGR-ALKBH5-siRNA-BNVs were administered to mice via intravenous injection. Mice body weight, blood routine, blood biochemistry markers (such as ALT, AST, BUN, Cr), and histopathological changes in major organs were monitored. The results showed no significant changes in body weight after injection (Figure S1A). Blood biochemistry markers were within normal ranges, with no significant abnormalities (Figure S1B). Histopathological examination revealed no significant pathological damage in major organs such as the heart, liver, spleen, kidneys, and lungs (Figure S1C), indicating that NGR-ALKBH5-siRNA-BNVs have good biocompatibility *in vivo*.

**Figure 5:**
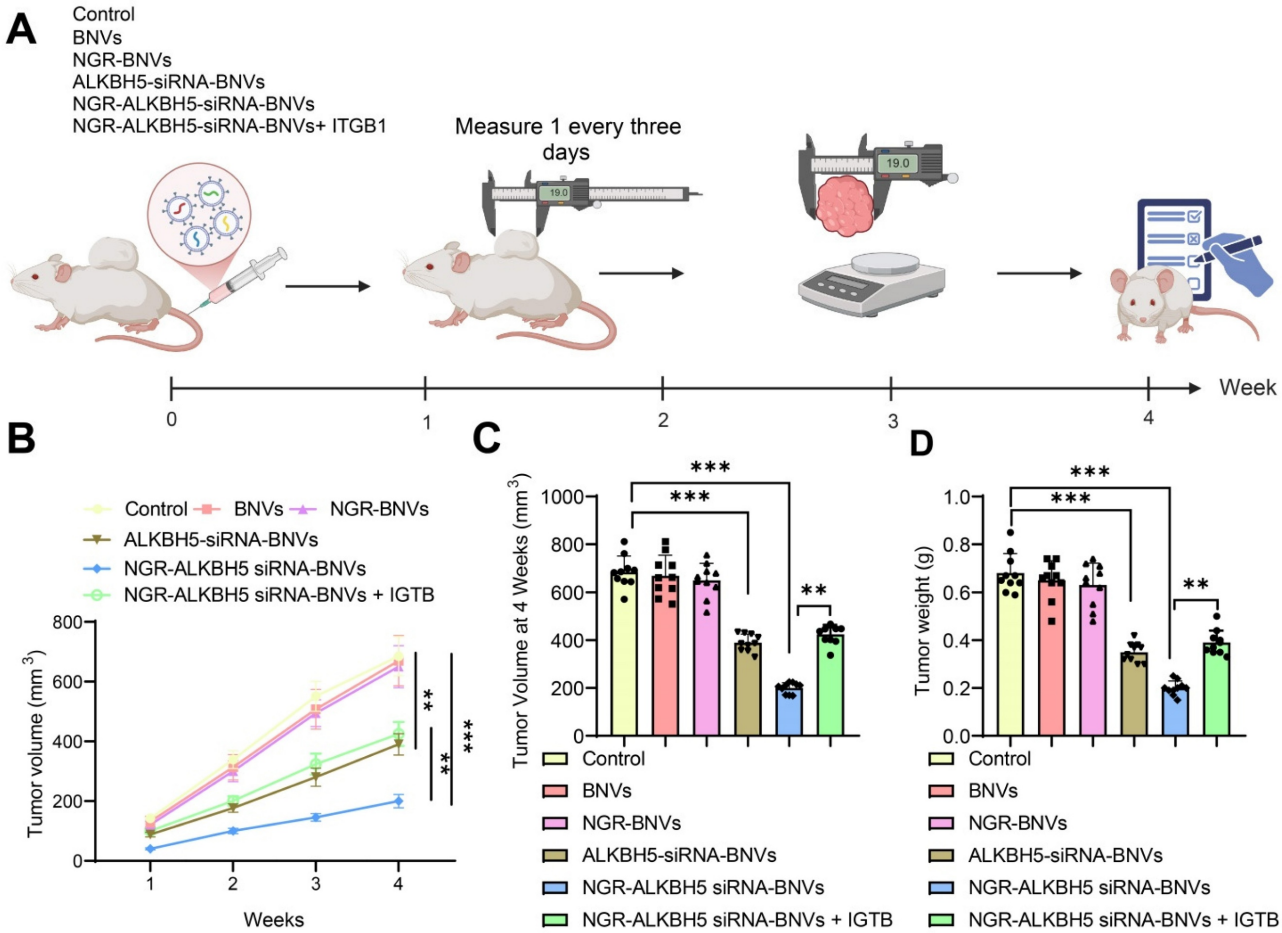
** Therapeutic Effect of NGR-Modified BNVs on OC Mouse Model.** Note: (A) Schematic diagram of mouse group intervention; (B) Tumor volume change curves of mice in the Control group, BNVs group, NGR-BNVs group, ALKBH5-siRNA-BNVs group, NGR-ALKBH5-siRNA-BNVs group, and NGR-ALKBH5-siRNA-BNVs + ITGB1 group during treatment; (C) Statistical analysis of tumor volume in each group; (D) Statistical analysis of tumor weight in each group during treatment. Each group consisted of 10 mice. Data in the graph are represented as mean ± SD. Statistical analysis was performed using ANOVA and Tukey's post hoc test, with * indicating *p* < 0.05, ** indicating *p* < 0.01, and *** indicating *p* < 0.001.

The schematic diagram of the intervention protocol in mice is shown (Figure 5A). In this study, the mice were randomly divided into six groups: Control group, BNVs group, NGR-BNVs group, ALKBH5-siRNA-BNVs group, NGR-ALKBH5-siRNA-BNVs group, and NGR-ALKBH5-siRNA-BNVs + ITGB1 group. Tumor volume was measured weekly, and changes were recorded.

The tumor volume and mass for each group of mice during the treatment period are presented (Figures 5B, C, and D). The results indicate that during the 4-week treatment period, compared to the Control group, tumor volume and mass were significantly reduced in the ALKBH5-siRNA-BNVs group and NGR-ALKBH5-siRNA-BNVs group, with the NGR-ALKBH5-siRNA-BNVs group showing more pronounced inhibition. Compared to the NGR-ALKBH5-siRNA-BNVs group, tumor volume and mass were significantly increased in the NGR-ALKBH5-siRNA-BNVs + ITGB1 group (p < 0.05). These results suggest that NGR-modified biomimetic nanovesicles carrying ALKBH5 siRNA significantly inhibit tumor growth by regulating ITGB1.

**Figure 6:**
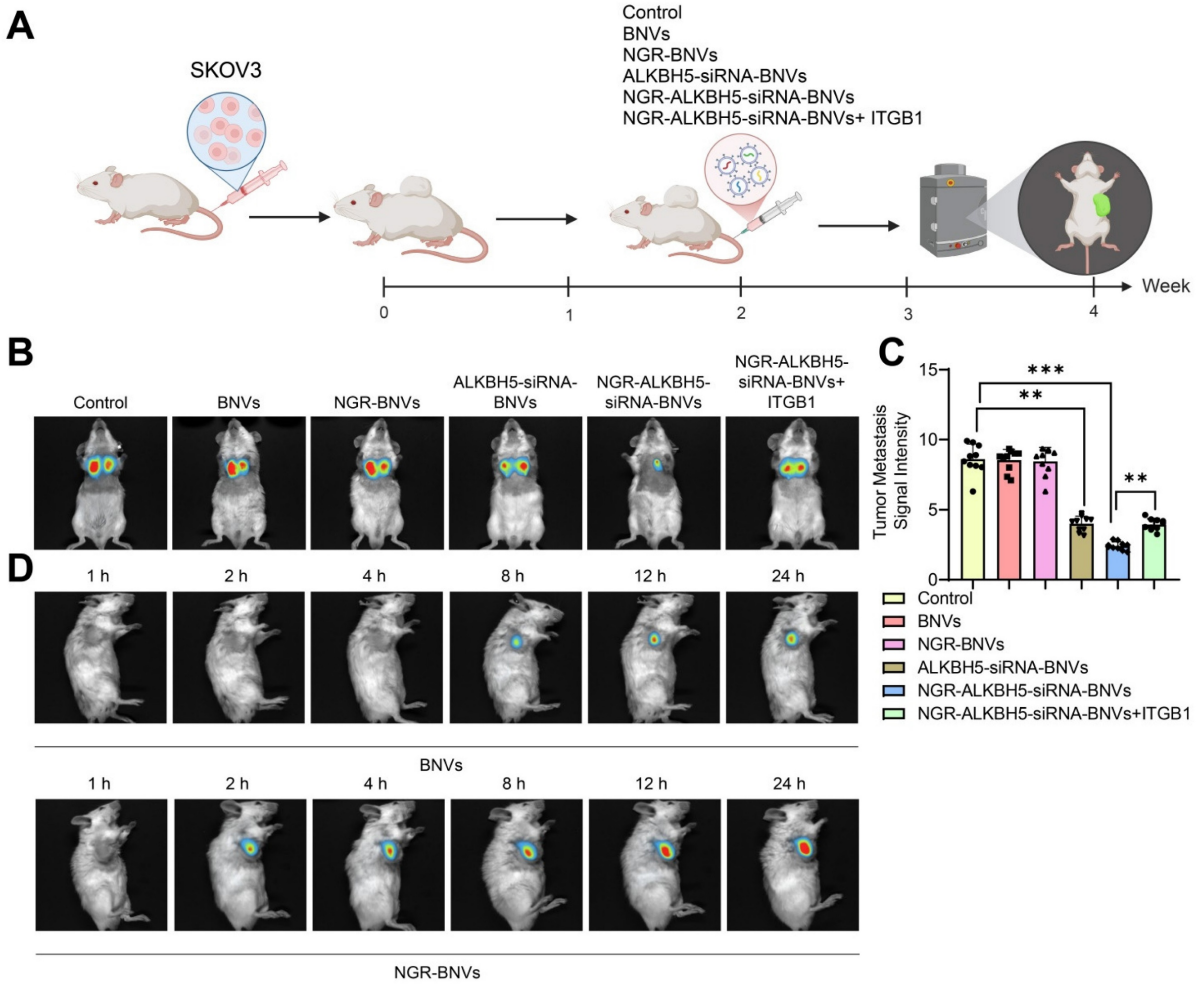
** BLI Analysis of the Impact of NGR-Modified BNVs on Tumor Metastasis in Mice.** Note: (A) Schematic representation of the process of NGR-modified BNVs treatment in tumor-bearing mice; (B) BLI showing differences in tumor metastasis signals in the treatment group, Rescue group, and control group mice; (C) Quantification of tumor metastasis signal intensity in each group; (D)* In vivo* distribution of nanovesicles observed at different time points (1, 2, 4, 8, 12 and 24 hours). The experiment was repeated three times with 10 mice per group. Data in the figures are presented as mean ± SD, and statistical analysis was conducted using ANOVA and Tukey post hoc test, where * indicates *p* < 0.05, ** indicates *p* < 0.01, and *** indicates *p* < 0.001.

The schematic diagram of the treatment process using NGR-modified BNVs in tumor-bearing mice is depicted (Figure 6A). Tumor metastasis signals in the mice were monitored using BLI technology. The BLI results and quantification of tumor metastasis signals in mice from different groups are shown (Figures 6B and C). The data clearly demonstrate that the tumor metastasis signals in the ALKBH5-siRNA-BNVs and NGR-ALKBH5-siRNA-BNVs groups were significantly lower than in the Control group. In contrast, the tumor metastasis signals in the NGR-ALKBH5-siRNA-BNVs + ITGB1 group were partially restored. Using an *in vivo* imaging system, the distribution of the nanovesicles in the mice was observed at different time points (1, 2, 4, 8, 12 and 24 hours). It was found that NGR-modified biomimetic nanovesicles effectively increased the accumulation and targeting at the tumor site (Figure 6D).

In conclusion, this study successfully inhibited tumor metastasis in mice using NGR-modified BNVs loaded with ALKBH5 siRNA, leading to a significant reduction in tumor metastasis signals. Particularly, the Treatment group, which received NGR-modified BNVs loaded with ALKBH5 siRNA alone, exhibited the most pronounced inhibition of tumor metastasis signals. These findings lay a solid foundation for further exploring the application of NGR-modified BNVs loaded with ALKBH5 siRNA in the treatment of OC.

### NGR-Modified BNVs Improve Tumor Immunosuppressive Microenvironment in Tumor-Bearing Mice

**Figure 7:**
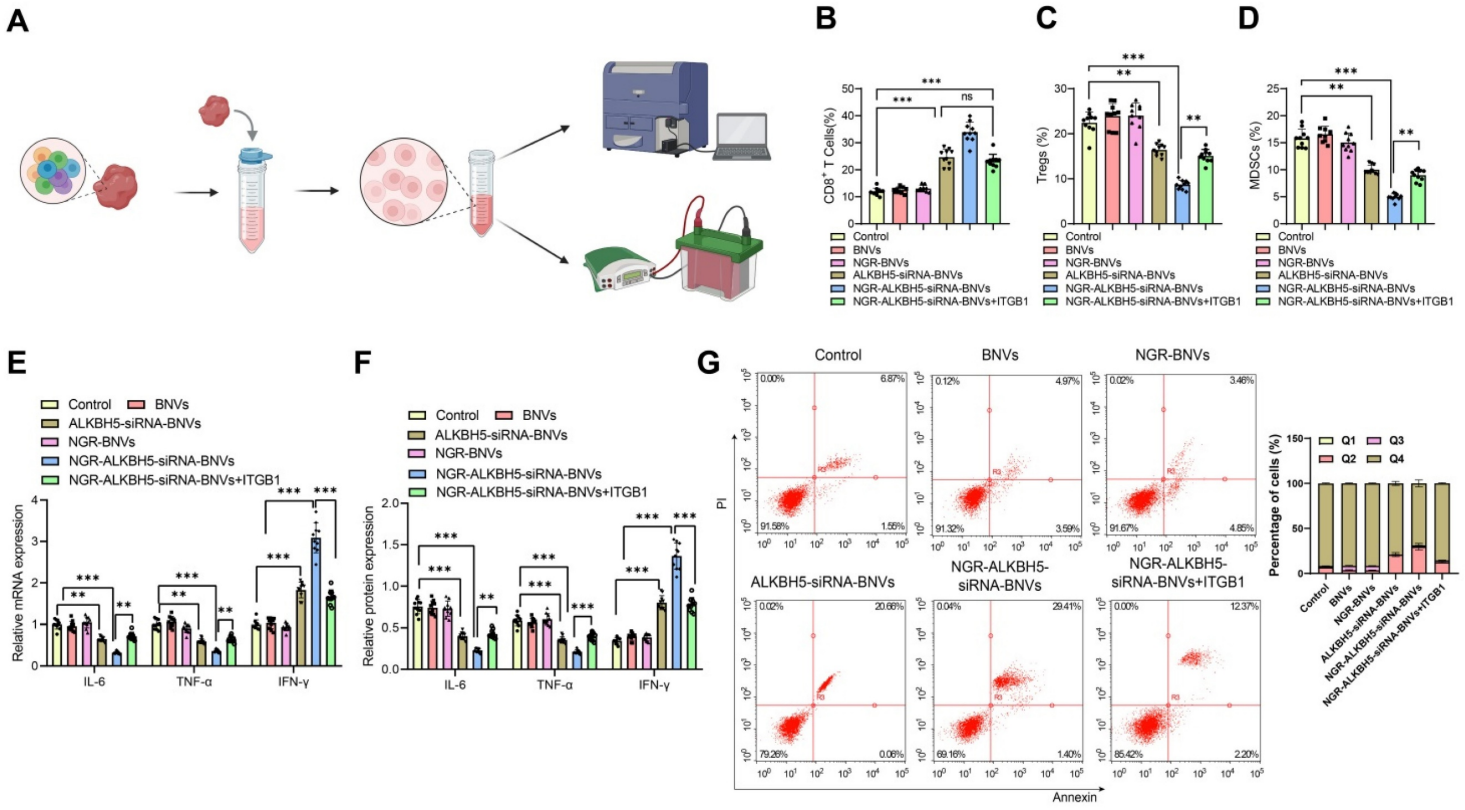
** The Impact of NGR-Modified BNVs on the TIME in Mice.** Note: (A) Schematic representation of TIME analysis process; (B) Percentage of CD8^+^ T cells in tumor tissue. Flow cytometry analysis revealed a significant increase in the proportion of CD8^+^ T cells in the tumor tissue of mice treated with the therapeutic and Rescue groups; (C) Percentage of Tregs in tumor tissue. Flow cytometry analysis showed a significant decrease in the proportion of Tregs in the tumor tissue of mice in the treatment and Rescue groups; (D) Percentage of MDSCs in tumor tissue. Flow cytometry analysis indicated a significant decrease in the proportion of MDSCs in the tumor tissue of mice in the treatment and Rescue groups; (E) mRNA expression levels of immune regulatory factors in tumor tissue. RT-qPCR detected the mRNA expression levels of immune regulatory factors such as IL-6, TNF-α, and IFN-γ in tumor tissue; (F) Protein expression levels of immune regulatory factors in tumor tissue. Western blot analysis measured the protein expression levels of immune regulatory factors like IL-6, TNF-α, and IFN-γ in tumor tissue; (G) Flow cytometry analysis of the proportion of apoptotic cells in tumor tissues from different groups of mice. Each group consisted of 10 mice. Data in the figures are presented as mean ± SD. Statistical analysis was performed using ANOVA and Tukey's post hoc test; * indicates *p* < 0.05, ** indicates *p* < 0.01, and *** indicates *p* < 0.001.

As shown in Figure 7A, the schematic diagram for immune microenvironment analysis. Flow cytometry analysis revealed that, compared to the Control group, the proportion of CD8+ T cells in tumor tissues was significantly increased in the ALKBH5-siRNA-BNVs and NGR-ALKBH5-siRNA-BNVs groups, while the proportions of Tregs and MDSCs were significantly reduced. Compared to the NGR-ALKBH5-siRNA-BNVs group, the proportion of CD8+ T cells was significantly reduced in the NGR-ALKBH5-siRNA-BNVs + ITGB1 group (P < 0.001, Figure 7B), while the proportions of Tregs and MDSCs were significantly increased (P < 0.001, Figure 7B-D).

RT-qPCR and Western blot results further validated this finding. Compared to the Control group, the ALKBH5-siRNA-BNVs and NGR-ALKBH5-siRNA-BNVs groups had significantly lower IL-6 and TNF-α mRNA and protein levels, while IFN-γ mRNA and protein levels were significantly increased. Compared to the NGR-ALKBH5-siRNA-BNVs group, the NGR-ALKBH5-siRNA-BNVs + ITGB1 group showed significant increases in IL-6 and TNF-α mRNA and protein levels, while IFN-γ mRNA and protein levels were significantly decreased (P < 0.001, Figure 7E-F).

In this study, the proportion of apoptotic cells in tumor tissues of mice was analyzed using flow cytometry. The results showed that, compared to the Control group, the ALKBH5-siRNA-BNVs and NGR-ALKBH5-siRNA-BNVs groups had significantly increased apoptosis in tumor tissues, confirming the efficacy of this treatment in inducing tumor cell apoptosis. Compared to the NGR-ALKBH5-siRNA-BNVs group, the proportion of apoptotic cells was significantly reduced in the NGR-ALKBH5-siRNA-BNVs + ITGB1 group (Figure 7G). These results provide important evidence for further exploring the application of NGR-modified biomimetic nanovesicles in cancer therapy.

In conclusion, the combined treatment of ALKBH5 siRNA and IGTB1 overexpression vector loaded in NGR-modified BNVs significantly inhibited tumor growth in mice. Furthermore, through immune microenvironment modulation, it enhanced anti-tumor immune responses in mice. This study provides essential experimental evidence for further exploring the application of NGR-modified BNVs in tumor immunotherapy.

## Discussion

OC is one of the most lethal malignancies in the female reproductive system, with high recurrence rates and issues with immunotherapy resistance severely limiting treatment efficacy 55-57. Recent research on immune checkpoint inhibitors has brought new hope for OC treatment, but immunotherapy resistance remains a widespread problem, necessitating new strategies to address this issue 58-60. Previous studies have shown that ALKBH5 plays a crucial role in m6A RNA modification and is closely associated with tumor progression and treatment resistance in various cancers 61-63. This study aims to reverse immunotherapy resistance in OC by using NGR-modified BNVs to deliver ALKBH5 siRNA and to explore its regulatory mechanism on ITGB1 m6A modification and its impact on the TIME. The innovation of this strategy lies in the combination of nanotechnology and RNA interference, aiming to provide new insights and methods for the treatment of OC 64-66.

In this study, we successfully prepared NGR-modified BNVs and conducted detailed characterization analyses. NGR peptides specifically target tumor vascular endothelial cells, enhancing the efficiency of ALKBH5 siRNA delivery to tumor sites. *In vitro* experiments demonstrated that NGR-modified nanovesicles significantly improved ALKBH5 siRNA delivery efficiency, reduced OC cell proliferation, and induced apoptosis 67-69. Additionally, *in vivo* experiments with a mouse model confirmed the therapeutic efficacy of this nanovesicle system, showing a significant reduction in tumor volume and a substantial increase in survival rate in the treatment group. Compared to traditional siRNA delivery systems, NGR-modified BNVs exhibited superior delivery efficiency, targeting specificity, and therapeutic effectiveness 70, 69.

This study validated the effectiveness of NGR-modified BNVs in delivering ALKBH5 siRNA through *in vitro* experiments. The nanovesicles significantly inhibited the proliferation of OC cells and induced apoptosis. Compared to other studies utilizing siRNA or nanovesicle technologies, our delivery system demonstrated superior efficiency and specificity. Previous research has shown that high ALKBH5 expression is closely associated with the resistance and invasiveness of various cancers; however, the specific regulatory mechanisms of its m6A modification remain unclear 22. By interfering with ALKBH5 expression, this study further explored its role in OC, revealing that ALKBH5 may regulate ITGB1 m6A modification, thereby affecting tumor cell proliferation and apoptosis. These findings provide new insights into the function of ALKBH5 in tumors.

*In vivo* experimental results demonstrate that NGR-modified BNVs are highly effective in delivering ALKBH5 siRNA. The treated mice exhibited significantly reduced tumor volume, decreased metastatic signals, and extended survival periods. Compared to other nanoparticle drugs or siRNA delivery systems tested *in vivo*, our nanovesicle system shows superior therapeutic efficacy and lower side effects. Previous studies have seldom explored *in vivo* interventions targeting ALKBH5. This study is the first to verify the feasibility and effectiveness of using NGR-modified BNVs to deliver ALKBH5 siRNA in reversing OC immunotherapy resistance, laying the groundwork for future clinical applications.

The TIME plays a crucial role in tumor progression and therapeutic resistance 71-73. Our study found that NGR-modified BNVs delivering ALKBH5 siRNA significantly altered the TIME. Specifically, there was an increase in the proportion of CD8+ T cells and a decrease in the proportions of Tregs and MDSCs in the tumor tissues of the treatment group mice. Additionally, there were significant changes in the expression levels of immune regulatory factors such as IL-6, TNF-α, and IFN-γ. The above changes indicate that NGR-modified biomimetic nanovesicles not only directly inhibit tumor cell proliferation and apoptosis and increase cancer cell sensitivity to T cells to reduce tumor cell numbers but also regulate the TIME, enhancing the anti-tumor immune response. We believe these two actions are interdependent, but immune regulation may have greater clinical significance. This finding provides new insights into the regulatory mechanisms of the TIME.

The high efficiency and specificity of NGR-modified BNVs for delivering ALKBH5 siRNA provide strong support for their potential clinical application. This strategy shows significant advantages in reversing resistance and enhancing efficacy, particularly in OC immunotherapy. Future research will focus on optimizing the design of these nanovesicles to improve delivery efficiency and targeting specificity. Additionally, more preclinical studies are needed to verify their application potential in other types of cancer. As nanotechnology and RNA interference technology continue to advance, this strategy is expected to play an increasingly important role in cancer treatment.

Despite the significant results achieved in this study, there are still some limitations. For example, the sample size is relatively small, and the study duration is short, which limits the comprehensive evaluation of the long-term safety and efficacy of the nanovesicles. Furthermore, due to physiological and pathological differences between animal models and human tumors, the feasibility of translating these results to clinical applications requires further validation. Future research should increase the sample size, extend the study period, and conduct more preclinical and clinical trials to fully assess the safety and efficacy of this strategy.

Previous studies have shown that ALKBH5 inhibits the YTHDF2 protein-mediated m6A-dependent degradation of ITGB1 mRNA, leading to increased ITGB1 expression and the phosphorylation of focal adhesion kinase and Src oncoproteins, thereby promoting lymph node metastasis 63. NGR is a peptide containing the arginine, glycine, and asparagine sequence and is a high-affinity ligand for CD13. In earlier studies, NGR peptides were used in combination with anti-cancer drugs to target tumor neovasculature. NGR-labeled nanoparticles can be used as a new platform for the delivery of single or multiple cytokines to tumor endothelial cells for cancer therapy 67, 74, 75. Combining previous studies with the* in vitro* and* in vivo* results from this research, we hypothesize that NGR-labeled ALKBH5 siRNA may inhibit ITGB1 expression by promoting the m6A-dependent degradation of ITGB1 mRNA mediated by YTHDF2, thereby participating in OC immune therapy resistance and optimizing the TIME to inhibit breast cancer progression. However, due to time and funding limitations, we have not further validated the regulatory mechanism of ALKBH5 on ITGB1. This will be further explored in future studies.

In conclusion, this study successfully utilized NGR-modified BNVs to deliver ALKBH5 siRNA, reversing immunotherapy resistance in OC, significantly inhibiting tumor growth and metastasis, and enhancing anti-tumor immune responses by modulating the TIME. The scientific and clinical value of this strategy lies in its efficient delivery system and significant therapeutic effects, providing new insights and methods for OC treatment. However, there are limitations that need to be addressed in future studies. Due to time and funding constraints, we were unable to include traditional chemotherapy or immunotherapy groups. We acknowledge this limitation and plan to address it in future studies in order to provide a more comprehensive assessment of the therapeutic effects of NGR-ALKBH5-siRNA-BNVs. Furthermore, with continued research, NGR-modified BNVs for delivering ALKBH5 siRNA are expected to play an increasingly pivotal role in cancer.

## Supplementary Material

Supplementary figure.
