# Supplementary material for: NGR-modified nanovesicles target ALKBH5 to inhibit ovarian cancer growth and metastasis
Source: Theranostics. 2025 Jun 9;15(14):6702–18. doi: 10.7150/thno.107766 (PMC12203674; doi:10.7150/thno.107766)
Supplement: Supplementary file 1 — Supplementary figure. [file thnov15p6702s1.pdf]

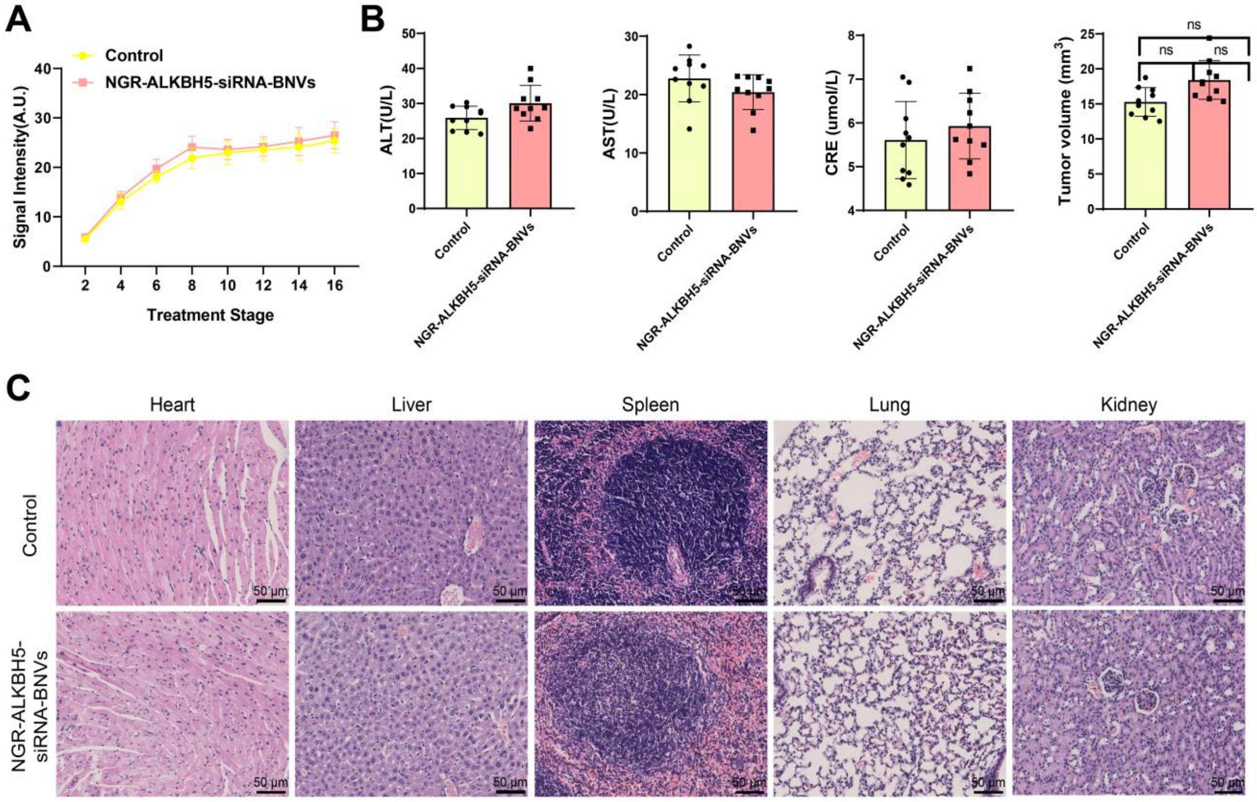

Figure S1. ***In vivo*** **biocompatibility of NGR-ALKBH5-siRNA-BNVs.**

Notes: (A) Monitoring of body weight changes in mice after intravenous injection of NGR-ALKBH5-siRNA-BNVs; (B) Blood biochemical analysis detecting ALT, AST, BUN, and Cr levels in mouse serum; (C) Histopathological examination analyzing pathological changes in major organs such as the heart, liver, spleen, kidneys, lungs, and renal tissues.
